# Determination of the Excitatory Effects of MicroRNA-30 in the Self-Renewal and Differentiation Process of Neonatal Mouse Spermatogonial Stem Cells

**DOI:** 10.31661/gmj.v9i0.1829

**Published:** 2020-08-19

**Authors:** Maryam Khanehzad, Farid Abolhasani, Gholamreza Hassanzadeh, Seyed Mehdi Nourashrafeddin, Azim Hedayatpour

**Affiliations:** ^1^Department of Anatomy, School of Medicine, Tehran University of Medical Sciences, Tehran, Iran; ^2^Department of Obstetrics, Gynecology and Reproductive Sciences, School of Medicine, University of Pittsburgh, Pittsburgh, USA; ^3^School of Advanced Technologies in Medicine, Tehran University of Medical Sciences, Tehran, Iran

**Keywords:** Adult Germline Stem Cell, MicroRNA, Cell Differentiation, Self-renewal

## Abstract

**Background:**

Spermatogonial stem cells (SSCs) are considered as special stem cells since they have the ability of self-renewal, differentiation, and transferring genetic information to the next generation. Also, they considered as vital players in initiating and preserving spermatogenesis. The fate decisions of SSCs are mediated by intrinsic and extrinsic factors, among which microRNAs (miRNAs) are one of the most essential factors in spermatogenesis among endogenous regulators. However, the mechanisms by which individual miRNAs regulate self-renewal and differentiation of SSCs are unclear. The present study aimed to evaluate the impact of miRNA-30 mimic on fate determinations of SSCs.

**Materials and Methods:**

The obtained SSCs from neonatal mice (3-6 days old) were purified by MACS and flow cytometry with a promyelocytic leukemia zinc-finger marker. Then, the cultured cells were transfected with miRNA- 30 mimic, and finally, the changes in expressing ID4 and c-kit proteins were assessed by western blot analysis.

**Results:**

According to flow cytometry findings, the percentage of SSC purity was about 98.32. The expression of ID4 protein and colonization increased significantly through the transfection of miRNA-30 mimic (P<0.05).

**Conclusion:**

The miRNA-30 controls spermatogonial stem cell self-renewal and differentiation, which may have significant implications for treating male infertility.

## Introduction

Spermatogenesis is an intricate and highly-developed process in the mammalian reproductive system that results in generating highly specialized sperm from spermatogonial stem cells (SSCs) [1,2]. However, SSCs play a vital role in spermatogenesis, which forms about 0.02–0.03% cells of the total cell population in the adult mouse testis [3,4]. SSCs are known for their capacity for the transition of genetic information to the next generations, self-renewal, and differential ability throughout life and pluripotency capability. Based on these specifications, SSCs could be utilized as an excellent plan to clarify the signaling pathways and genetic modification experiments [5,6]. Also, they could be applied for cell therapy, regenerative therapy, and infertility treatment [7]. The equivalence between SSCs and spermatocytes is essential for initiating and maintaining normal spermatogenesis. This balance is regulated by both intrinsic and extrinsic agents [8,9]. Some recent studies introduced a class of endogenous factors called microRNAs (miRNAs), which play a significant role in diverse biological processes such as self-renewal, proliferation, differentiation, and apoptosis [10]. The miRNAs-small single-stranded RNA molecules (18–i.e., 25 nucleotides) are considered as vital factors in the post-transcriptional gene silencing. They bind with the three untranslated regions of target mRNAs and, accordingly, either endonucleolytic cleavage of the target mRNA or inhibition of translation [8,11]. Some studies reported miRNAs as an important regulatory factor in spermatogenesis [12,13]. For example, the high expression of miRNA-100 in SSCs promotes SSCs proliferation by STAT3 [13]. Further, the self-renewal of SSCs is regulated by miRNA -10b and miRNA -322 [4]. Furthermore, the inhibition of cell cycle regulators and RNA binding proteins by miRNA -202 result in maintaining SSCs [14]. Additionally, miRNA‐34c increases the differentiation of mouse SSCs by targeting Nanos2 [15]. In addition, miRNA -17-92 (Mirc1) and miRNA -106b-25 (Mirc3) play a key role in promoting spermatogonial differentiation in mice [9]. The differentiation of spermatogonia via RA leads to the suppression of LIN28 and induction of the Mirlet7 family, which can downregulate the genes associated with the self-renewal of spermatogonia [16]. Nevertheless, the function and molecular mechanisms of individual miRNAs in regulating determining SSCs fate are ambiguous, which necessitates further research in this area. A large number of studies evaluated the miRNA-30 family effects and reported that miRNA-30 could regulate cell proliferation and differentiation processes in different cells. Further, the developmental process of tissues and organs, as well as the related diseases, can be influenced by the miRNA-30 family [17-19]. For example, the inhibition of the miR-30 family led to a significant decrease and increase in proliferating and differentiating intestinal epithelial cells, respectively. Furthermore, a significant decrease in miR-30 expression was reported in differentiating mesenchymal stem cells and C2C12 induced by bone morphogenetic protein 2 [20,21]. On the other hand, the presence of miR-30 as an important regulator was detected in various cell lines such as progenitor germ cells, SSCs, and mouse and human testis tissue [22-24]. Based on the presented documents, miRNA-30 seems critical for male fertility and reproductive development. However, the effects of miR-30 in determining SSCs’ fate are not clear, which necessitates further research in this area. Thus, the present study sought to define miRNA- 30 mimic effects in self-renewing and differentiating mouse SSCs.

## Materials and Methods

###  Animals

 BALB/c male mice (3-6 days old) were purchased from Faculty of Pharmacy in Tehran University of Medical Sciences. All in-vivo studies were performed based on standard operational procedures and regulation by the Ethics Committee of Tehran University of Medical Sciences (IR.TUMS.MEDICINE.REC.1396.3940).

###  Isolation of SSCs

In this section, the protocol of Kanatsu-Shinohara with slight modification was used for isolating SSCs [25]. First, anesthesia was performed with 0.05 mg/kg ketamine (Sigma-Aldrich, St. Louis, MO). Then, the removed testes were rapidly transferred into culture dishes, including fresh phosphate-buffered saline (PBS, Sigma-Aldrich.USA). Under sterile conditions, the PBS supplemented with 1% pen/strep was used for washing the samples. During enzymatic digestion, the minced samples were put into digestion medium containing 5µg/mL DNase (Sigma-Aldrich.USA), 1 mg/mL collagenase type IV (Gibco,CA), and 1mg/mL hyaluronidase (Sigma-Aldrich. USA), which were incubated for 20 min at 37°C with 5% CO2. The suspension was pipetted gently every 5 minutes. In addition, the centrifuge was done at 1500 g for 5 minutes. In the next step, the same digestion medium was utilized for purifying cellular pellets for 15 minutes. Finally, the viability of cells was evaluated using 0.04 % trypan blue and hemocytometer.

###  Immunocytochemistry

 The immunocytochemistry with specific marker vimentin was used for verifying Sertoli cells. First, SSCs were fixed with paraformaldehyde 4% (Sigma-Aldrich) and permeabilized with 0.1% Triton X-100 (Sigma-Aldrich) followed by blocking for one hour with 10% goat serum (Sigma-Aldrich). Then, the samples were incubated with mouse monoclonal anti-Vimentin antibody (Sigma v-6630) for 24 hours, followed by 2-hour exposure with secondary antibody fluorescein isothiocyanate (FITC; ab6717, Abcam, UK). In addition, nuclei were stained with 4¢, 6-diamidino-2-phenylindole (DAPI, 1 lg/mL). Finally, the slides were observed by a fluorescence microscope (Olympus LX71, Japan)

###  Enriching SSCs

Initially, somatic cells were separated by differential plating for enriching SSCs. In this method, Petri dishes were coated with 5 μg/ml Lectin (Sigma-L2766.USA). Then, enzymatic digestion suspension was added to the dish. The somatic cells were attached to the dish after an hour, and the supernatant containing SSCs was collected [26,27]. Further, promyelocytic leukemia zinc finger (PLZF)-positive cells were purified by magnetic-activated cell sorting (MACS) with magnetic microbeads conjugated to an anti-PLZF antibody (Abcam, Cambridge, MA, www.abcam.com), as it was previously described [6,28,29]. Furthermore, flow cytometry was conducted with a PLZF marker in order to specify the purity of SSCs. Brieﬂy, 105 cells were incubated in 100 µl PBS/ fetal bovine serum (FBS) and 10 µl primary antibody PLZF (30 min, 4°C). Then, the cells were washed twice in 1 mL of PBS/ FBS. In the next procedure, 100 µl PBS/FBS and 10 µl secondary antibody (FITC) were added (20 min, 4°C). However, no antibody was added to the control group. Finally, the cells were preserved in the darkroom on the ice pack, and the purity percentage of the cells was defined by ﬂow cytometry.

###  Culturing SSCs

 In order to culture, the purified cell (1×105 cell/cm2) medium containing Dulbecco’s modified Eagle’s medium (DMEM) was replaced with 10% FBS (Life Technologies), 10 ng/Ml leukemia inhibitory factor (LIF; Sigma, Haverhill), 10 ng/mL basic fibroblast growth factor (Peprotech, Rocky Hill, NJ), 0.1 mM β-mercaptoethanol (Sigma-Aldrich), 10 μg/mL glial cell line-derived neurotrophic factor (GDNF; Sigma-Aldrich. USA), 100 U/mL penicillin (Sigma-Aldrich, Darmstadt), and 100 μg/mL streptomycin (Sigma, Germany). Then, all of the cultures were incubated at 37°C in a humidiﬁed 5% CO incubator and the medium was refreshed every 2-3 days.

###  Transfecting miR-30 Mimic and miR-30 Inhibitor into Mouse SSCs

First, miRNA-30 mimic (HMI0454) was provided from Sigma-Aldrich. Then, dosimetry experiments were accomplished for optimizing miR-30 mimic concentration. Eventually, 100nM was attained as an optimal dose. In the next procedure, miR-30 mimic was diluted in 125µl Opti-MEM (Invitrogen) reduced serum medium (Cat. No. 31985-062). Then, the mixed medium was incubated for 5 minutes at room temperature (RT). At the same time, 1 µl Lipofectamine 2000 transfection agent (Invitrogen) was added to 50 µl Opti-MEM reduced serum medium and incubated for 5 minutes at RT. Further, diluted miR and lipofectamine were incubated for 20 min at RT. Finally, this medium was transfected to SSCs and incubated at 37°C. Additionally, the transfection medium was replaced 4 hours later by fresh growth medium, and the cells were collected for evaluating the changes in expressing the proteins after transfecting for 48 hours. In addition, the colonization of SSCs was assessed after one week [6,7,14]. Finally, the cultured SSCs were divided into four groups, including no miRNA -30 transfection, miRNA-30 mimics, miRNA-30 mimic control, and only lipofectamine.

###  Assessing SSCs Colonization

First, the diameter and number of colonies were determined by an inverted microscope (Olympus, CKX41, Japan). Then, the data were analyzed by Image J software (version 1.240; National Institutes of Health, Bethesda, MD, USA) [30].

###  Quantitative Real-Time Polymerase Chain Reaction (PCR)

 In the present study, TRIzol reagent (Invitrogen) was used for extracting the total RNA. First, the RNAs were reversely transcribed by System Kit (ZistRoyesh, Iran) according to the instructions of reagents. Then, the real-time PCR was performed with GoTaq qPCR Master Mix (Promega) on the Applied Biosystems 7500 Sequence Detection system. In addition, the SYBR Green was utilized as a detection medium. Further, the analysis of the melt curve was accomplished for identifying nonspecific PCR products and primer dimers. All of the tests were conducted at least three times. Additionally, the U6 snRNA was selected as an internal control gene for normalization. The 2-∆∆Ct method with reference to the expression of U6 snRNA was used for determining and normalizing gene expression levels. Table-1 indicates the specific primer sequences.

###  Western Blot

 A TriPure Isolation Reagent (Roche, Germany) was used for extracting the total protein from SSCs. Then, electrophoresis was employed to separate 20 µg of the total protein for each sample. In the next step, the proteins were transferred into 10.5% and 12.5% gradient sodium dodecyl sulfate-polyacrylamide gel (BioRad Laboratories, Hercules, CA) and polyvinylidene difluoride membranes (Roche, Germany), and then were blocked with 5% non-fat dry milk (Carnation, CA). Subsequently, the primary antibodies against ID4 and c-kit (1:1000) were used for incubating specimens for 24 hours at 4°C, and then secondary antibodies and HRP were added. Finally, the rates of protein expression were evaluated with enhanced chemiluminescence.

###  Statistical Analysis

 GraphPad Prism 7.0 (GraphPad Software, Inc., La Jolla, California), one-way ANOVA method, and Tukey post-hoc test were used for data analysis. The significance level of 0.05 was determined as significant.

## Results

###  Determining the Purification of SSCs 

**Figure 1:**
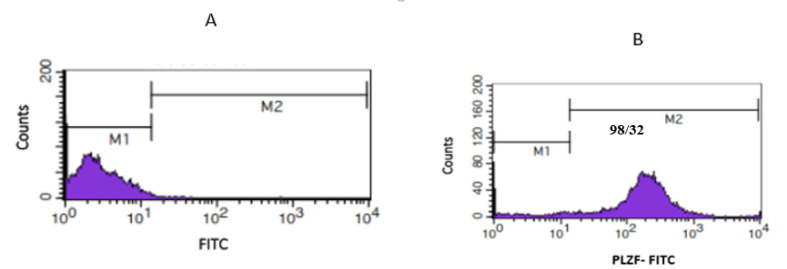
Results of flow cytometry display the percentage of purity of SSCs with marker PLZF (M1: PLZF-negative cells, M2: PLZF-positive cells. A: Control; B: PLZF positive)

 The PLZF gene, as an undifferentiated spermatogonia marker, was selected for identifying the SSCs. This marker was evaluated by flow cytometry. As shown in Figure-1, the percentage of expressions for this marker is 98.32% in the SSCs.

###  Identifying Sertoli Cells

**Figure 2:**
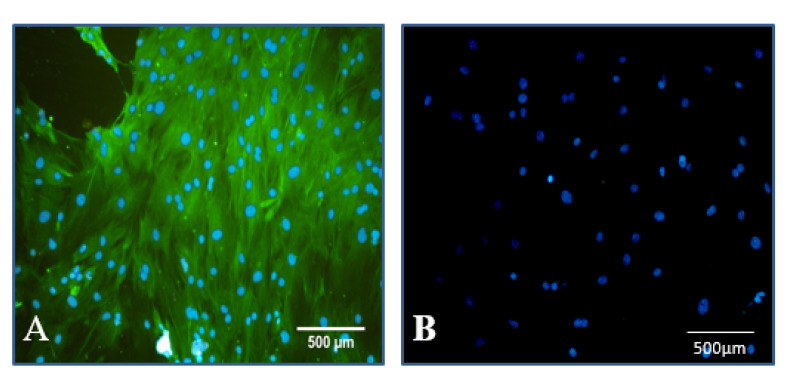
Identification of Sertoli cells by immunocytochemistry. Sertoli cells were positive for vimentin in the cytoplasm (green color), and nuclei were stained with DAPI (blue color, **A**) and negative control ( **B**).

 Immunocytochemistry with specific marker vimentin was used for verifying Sertoli cells. The obtained images confirmed Sertoli cells (Figure-2).

###  Colony Assay

**Figure 3:**
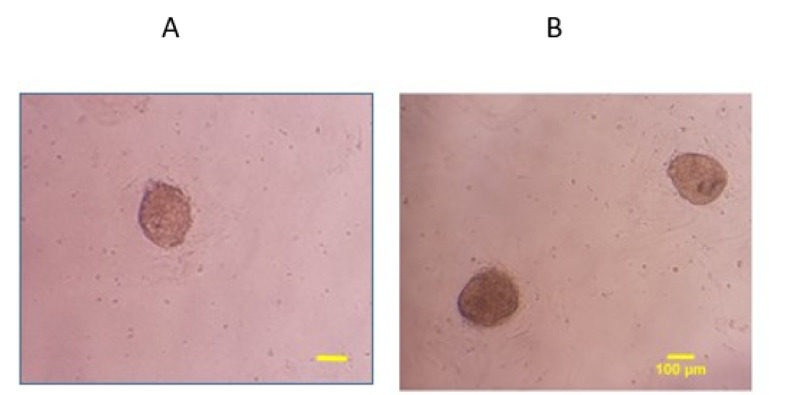
Microscopic morphology of SSCs colony. **A**: No miRNA transfection group; **B**: MiRNA-30 mimic group (Scale bar=100 μm).

**Figure 4:**
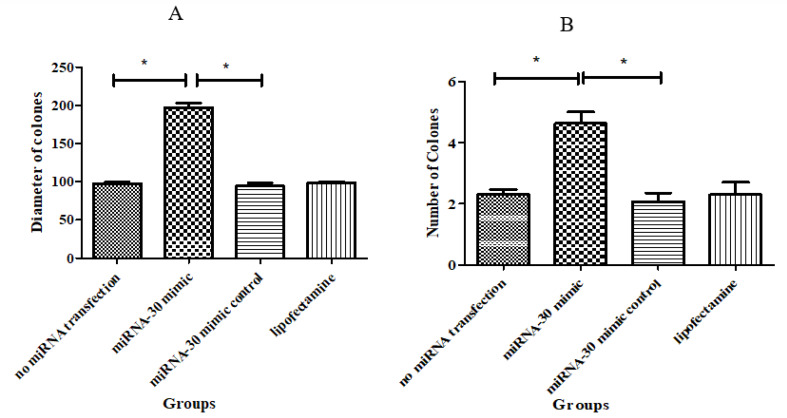
A comparison of colony diameter ( **A**) and ( **B**) numbers in study groups. Data are represented as means± SE, *P ≤ 0.05

 As displayed in Figure-3, the images of colonies were captured by an inverted microscope one week after transfection. As shown, the numbers (4.63±0.37) and diameters (197± 5.85μm) of colonies in miRNA-30 mimic significantly increased compared to other groups (P≤ 0.05, Figures-4).

###  Results of Quantitative Real-Time PCR 

**Figure 5:**
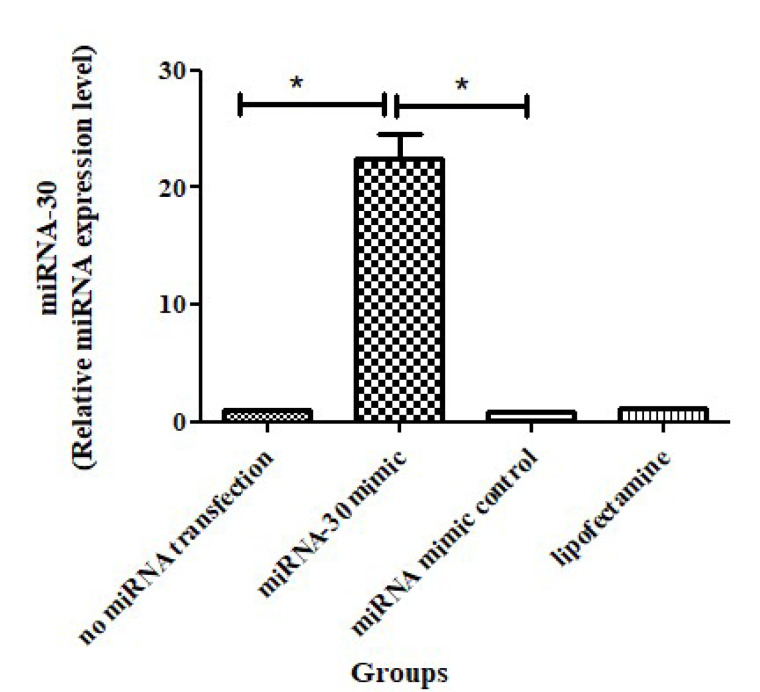
Transfection efﬁciency of miRNA-30 Mimic in SSCs, *P<0.05

 In this study, miRNA-30 mimic was utilized to evaluate the function of miRNA-30 in spermatogenesis. The q RT-PCR technique was used for demonstrating miRNA-30 mimic after determining the efficiency of transfection. Based on the results, the expression level of miRNA-30 significantly increased in the miR30 mimic group (Figure-5).

###  Results of Western Blot

**Figure 6:**
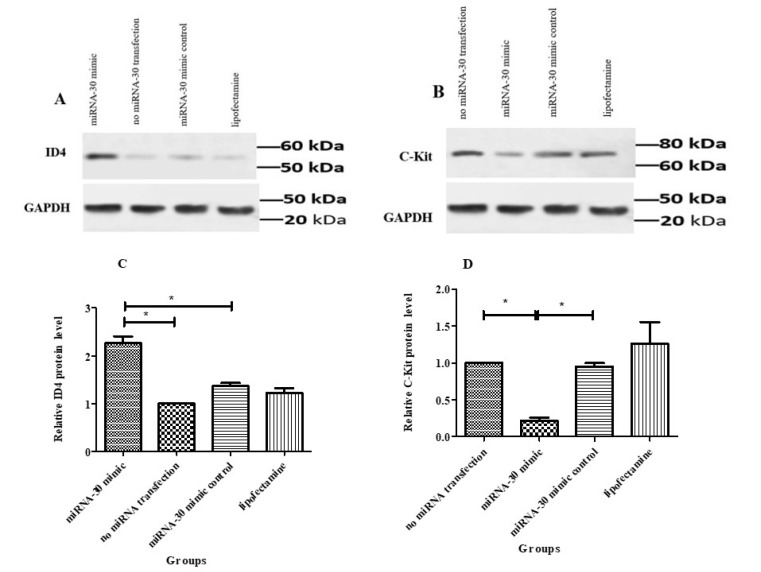
Western blot illustrates the changes of ID4 ( **A**) and c-kit ( **B**) proteins in transfected SSCs. GAPDH was utilized as the internal control. Graphs present the ratio for normalization of the density of ID4 ( **C**) and c-kit ( **D**) to the GAPDH. *P<0.05.

 In order to evaluate the effects of miRNA-30 in SSCs fate regulation, the changes in ID4 (undifferentiated marker) and c-kit (differentiated marker) proteins were examined by western blot. The findings of western blot indicated a considerable increase in the expression level of ID4 in SSCs under the exposure to miR-30 mimic (2.263± 0.14) compared to the other groups (Figures-6A and C). On the other hand, the transcription of c-kit protein decreased significantly (0.245±0.04) in miRNA-30 mimic (Figures-6B and D). Thus, miRNA-30 can be considered as a significant controller in regulating SSCs’ self-renewal and differentiation.

## Discussion

In the present study, SSCs were considered as foundation cells for normal spermatogenesis. The preservation of the balance between SSCs and spermatocytes is regarded as a necessary element for supporting and promoting spermatogenesis. There are many intrinsic factors and extrinsic signals which play a role in regulating the balance. In this regard, the detection of intrinsic regulators and their roles in determining SSCs fate is essential as preclinical studies [31,32]. The results of some studies indicated that miRNAs could regulate proliferation and differentiation processes in a wide range of cells. For example, miRNA -10b, miRNA -322, miRNA 202, miRNA 106, miRNA 21, and miRNA 34c play a significant role in proliferating and differentiating SSCs [4-6,14,15,33]. However, molecular mechanisms and the role of various miRNAs in regulating spermatogenesis are still ambiguous. Thus, the present study focused on the regulation of the self-renewal and differentiation processes in SSCs after exposure to miRNA -30 mimic. In the present study, neonatal mice (3-6 days) were used for isolating SSCs. First, SSCs emerged at 3-6 days postpartum. Then, SSCs started to colonize and differentiate the spermatocyte cell lines. Furthermore, there are only 0.02 to 0.03 SSCs per testis. Based on the presented information, selecting neonatal mice for obtaining SSCs with high purity seems reasonable. Some researchers indicated that neonatal mice have a high purity of SSCs [4,6,34]. Given the recent discovery about the coordinating role of miRNA expression in Sertoli cells in androgen-dependent spermatogenic events, the present study focused on different plating to separate Sertoli and somatic cells to evaluate only the effect of miRNA [35,36]. Also, the PLZF, as one of the most well-known surface marker, was utilized for purifying SSCs. The result of the flow cytometry indicated that 98.32 % of the cells could express PLZF. Other markers that were considered in the literature are foxo1, ID-4, Thy1, α6, β1 [5,37]. It is worth noting that no special marker has yet been recommended for purifying SSCs, although others declared this marker as an efficient SSC marker. In addition, 24–96 hours are considered as the best time for evaluating miRNA effects after transfecting lipofectamine. Thus, in the present study, 100 nM miRNA mimic was transfected into SSCs, and the expression of proteins was assessed 48 hours after transfection. In the previous studies, the various genes and proteins were evaluated 48 hours after transfecting miRNAs, which is similar to the technique used in our study [6,7,38]. The western blot of factor related to proliferation or undifferentiated marker showed that ID4 expression considerably increases in SSCs treated with the mimic. It seems that miRNA-30 can be considered as a potent inducer for regulating SSCs self-renewal. Further, Niu *et al*. introduced miRNA 21 as a critical factor for SSCs self-renewal. In another study, miR-322 overexpression resulted in increasing GFRα1, ETV5, and PLZF expression; and decreasing STRA8 and c-kit expression. Based on the results, miR-322 is considered as the main factor for regulating self- renewal of SSCs. Further, the results of Chen *et al*. study indicated that miRNA -202 preserves SSCs. The results of the present study are inconsistent with those of Yu *et al*., in which that miR-34c over-expression promotes the differentiation process by up-regulation of Stra8 in SSCs [4,5,14,15]. Additionally, c-kit is a member of the class III receptor tyrosine kinases, which is detected only in differentiated spermatogonia, but not in SSCs. Considering that c-kit is a well-characterized marker of spermatogonial differentiation [39], low expression of c-kit was observed in the mimic group in the present study. The result suggests that over-expression of miRNA-30 inhibits the differentiation of SSCs, which are not in line with those of Tong. They reported that Mir-17-92 and Mir-106b-25 clusters could contribute to the promotion of the differentiation process in spermatogonial stem cells among mice [9]. In addition, an increase occurred in colony diameters and numbers in the SSCs treated with miRNA-30 mimic that is consistent with the induction effect of miRNA in SSC proliferation. Further, similar results were obtained by different methods used by other researchers [4,6]. In sum, the present study was performed to reveal novel therapeutic strategies that may be suitable in clinical studies. Future studies can be designed to elucidate key signaling pathways in spermatogenesis, which can pave the way to design more effective treatment of male infertility.

## Conclusion

 Our study indicated the excitatory effect of miRNA-30 on self-renewing and proliferating SSCs. The findings may be used for designing promising therapeutic strategies in cases with male infertility.

## Conflict of Interest

 The authors declare no potential conflicts of interest.

**Table 1:** The Sequence of Primers

**Name of gene**	**Primers**	**Sequence (5’-3’)**
**miR-30**	Forward Primer	GCGTGTAAACATCCTCGAC
	Reverse Primer	GTGCAGGGTCCGAGGT
**U6**	Forward Primer	CTCGCTTCGGCAGCACA
	Reverse Primer	AACGCTTCACGAATTTGCGT
